# Factors affecting the shear wave elastic quantitative measurement of penile tissue in rats

**DOI:** 10.2478/abm-2023-0040

**Published:** 2023-08-07

**Authors:** Wan-Ting Rao, Jing-Dong Tang, Jin-Fang Xing

**Affiliations:** Department of Medical Ultrasound, Fudan University Pudong Medical Center, Shanghai 201399, China; Shanghai Key Laboratory of Vascular Lesions Regulation and Remodeling, Fudan University Pudong Medical Center, Shanghai 201399, China

**Keywords:** collagen fibers, penis, shear wave elastic quantitative measurement, smooth muscle cells, ultrasonography

## Abstract

**Background:**

As a new ultrasound technology, 2-dimensional shear wave elastography (2D-SWE) can evaluate the elastic characteristics of penile tissue. However, no studies have reported the main factors affecting the shear wave elastic quantitative measurement (SWQ) in penile tissue.

**Objectives:**

To analyze the main factors affecting the SWQ reflecting the elastic characteristics of penile tissue by 2D-SWE.

**Methods:**

Twenty healthy male Sprague–Dawley rats (5–60 weeks old) were selected for this study. We performed the 2D-SWE examination on the penis using the Aixplorer ultrasound system, with SWQ as the measurement index. We performed penile immunohistochemistry analysis with the positive area proportion (PAP) of alpha-smooth muscle actin (PAP_S_) and type III collagen fiber (PAP_C_) as the outcomes. Then, we conducted multiple linear regression analysis to explore the correlation of SWQ with PAP_S_ and PAP_C_ and established the regression equation.

**Results:**

The multiple linear regression analysis showed that the linear regression equation (SWQ = 10.376 – 0.05 PAP_S_ – 0.07 PAP_C_) was statistically significant (*F* = 21.153, *P* < 0.001). The content of smooth muscle cells (SMCs) and collagen fibers was negatively correlated with SWQ, affecting 42.6% of the total variation in SWQ (*R*^2^ = 0.426).

**Conclusions:**

SMCs and collagen fibers are the main factors affecting the SWQ value of penile tissue and the primary tissue components determining the SWQ when using 2D-SWE to quantitatively evaluate the elastic characteristics of penile tissue.

Two-dimensional shear wave elastography (2D-SWE) is a new ultrasound technology applied to quantitatively evaluate tissue elastic characteristics by obtaining the shear wave elastic quantitative measurement (SWQ). SWQ is a quantitative index reflecting the tissue elastic characteristics, which is calculated by measuring the velocity of shear waves generated from tissue [1]. The SWQ value is affected by the component and content of tissue that the shear waves pass through.

Preliminary studies have shown that 2D-SWE is a new technology that can evaluate the elastic characteristics of penile tissue in a noninvasive manner [2, 3]. It is necessary to determine which components are the main factors affecting the SWQ in penile tissue when using 2D-SWE to diagnose penile diseases. As there have been no related studies reported, this study aimed to analyze the main components affecting the SWQ value in penile tissue.

Smooth muscle cells (SMCs) and collagen fibers (CFs) are the main components in penile tissue and the primary tissue components involved in regulating the process of penile erection [4, 5]. Changes in SMCs and CFs (the degeneration and apoptosis of SMCs, the proliferation and accumulation of CFs, and changes in the SMCs/CFs ratio, etc.) are the histopathological basis of organic erectile dysfunction (ED). There are many causes of organic ED, such as aging, testosterone deficiency, hypertension, diabetes, and Peyronie's disease [PD]) [6]. Therefore, we proposed the hypothesis that the content of SMCs and CFs was one of the main factors affecting the SWQ value in penile tissue. As Sprague–Dawley rats are one of the most commonly used animal models in andrology, we selected healthy Sprague–Dawley rats for this study. We performed the 2D-SWE examination and immunohistochemistry on the penis to validate our hypothesis.

## Methods

### Experimental animals

Twenty healthy male Sprague–Dawley rats (5–60 weeks) that weighed 109.43–450.02 g were selected from the Animal Breeding Center of the Fudan University Pudong Medical Center (Shanghai, China). The rats were housed single-caged and maintained at 23 ± 2°C on a 12-h light/12-h darkness schedule, with *ad libitum* food and water. The adaptive feeding lasted for 1 week. As the animals were not grouped, we did not establish the criteria for excluding animals and performed no blinding or randomization during the experimental procedures. All animal experiments were approved by the Ethics Committee of Fudan University Pudong Medical Center (Registration number: WZ-008) and followed the ARRIVE guidelines 2.0 [7]. No adverse events were observed during the study.

### 2D-SWE examination

After 1 week of adaptive feeding, the rats were anesthetized by an intraperitoneal injection of pentobarbital sodium (30 mg/kg) in an ultrasonography room at room temperature and placed in the supine position with 4 limbs fixed. We performed the 2D-SWE examination by using the Aixplorer ultrasound system (SuperSonic Imagine, Aix-en-Provence, France) with an SL15–4 probe and conducted transverse scanning near the glans, middle, and root segments of the penis. After obtaining an optimal 2-dimensional grayscale image, we chose “SWE” mode and adjusted the real-time elastography map to cover the whole penis with 0–50 kPa as the scale.

We depicted the region of interest (ROI) along the tunica albuginea, with a diameter of 2–4 mm. The penile SWQ was considered to be measured successfully when the color filling in the ROI was complete without artifacts.

### Histological analysis

After the 2D-SWE examination, the rats were euthanized at humane endpoints (AVMA Guidelines for the Euthanasia of Animals: 2020 Edition) by an intraperitoneal injection of pentobarbital sodium (150 mg/kg). We separated the penile tissue for immunohistochemistry analysis. The penile tissue was fixed in 4.0% neutral formalin, embedded in paraffin, and sectioned at 5 μm. Immunohistochemical staining was performed on the sections. After deparaffinization, the sections were heated in a microwave (Midea Group Co., Ltd., Foshan, China) oven at 95°C for 10 min for antigen retrieval and endogenous peroxidase inactivation. The sections were then incubated with anti-alpha-smooth muscle actin antibody (1:50; Abcam, Cambridge, MA, USA; No. ab82247) and anti-collagen III antibody (1:50; Abcam, Cambridge, MA, USA; No. ab6310) at 4°C overnight and subsequently incubated with rabbit anti-rat secondary antibody (Abcam, Cambridge, MA, USA; No. ab6734) at room temperature for 15 min. Ultimately, the sections were treated with 3′3-diaminobenzidine (DAB), counterstained with hematoxylin, dehydrated in fractionated ethanol, cleared, and then fixed in xylene. The steps for the immunohistochemical staining of type III CFs were the same as above, except that the primary antibody was replaced by phosphate-buffered saline (PBS) as the negative control.

Three fields of each section were randomly selected to measure the proportion of the positive area, and the average was subsequently calculated. The measurement indexes were as follows: the positive area proportion (PAP) of alpha-smooth muscle actin (PAP_S_) and type III CF (PAP_C_).

### Statistical analysis

In this study, we used the SPSS 26.0 software (SPSS, Inc., Chicago, USA) for statistical analysis. Data were expressed as the mean ± standard deviation (SD) (X̅ ± sd). We performed the Shapiro–Wilk (S–W) test to determine whether SWQ, PAP_S_, and PAP_C_ data followed a normal distribution. If the data followed a normal distribution, we selected Pearson correlation analysis to evaluate whether there was a linear relationship between SWQ and PAP_S_, SWQ and PAP_C_, respectively; otherwise, we draw scatter plots to observe it. If a linear relationship existed, we performed the multiple linear regression analysis to explore the correlation of SWQ determined by 2D-SWE examination and PAP_S_ and PAP_C_ determined by immunohistochemistry analysis and established the regression equation. We used the tolerance and variance inflation factor (VIF) of collinearity statistics to determine the multicollinearity between PAP_S_ and PAP_C_ and performed the S–W test, Durbin–Watson statistic, and residual plots to determine the normality, independence, and equal variance of residuals. *P* < 0.05 was considered as statistically significant.

## Results

### 2D-SWE results

**Figure 1.**
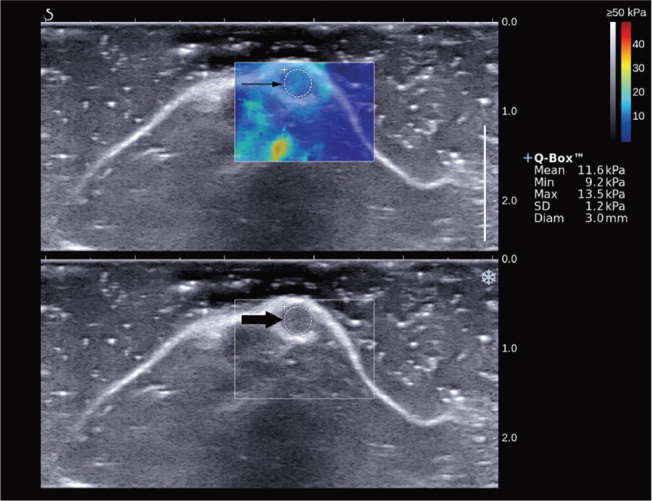
2D-SWE imaging of the penis. The grayscale image of the rat penis clearly showed that the cross-section of the rat penis (below, the O area indicated by the thick arrow) was oval, with a clear boundary, intact capsule, and uniform low echogenicity in the interior. The 2D-SWE image showed that the ROI within the rat penile tissue (above, the O area indicated by the thin arrow) was completely filled with color, similar to an oil painting without mosaic-like points. +Q-Box™, shear wave elastic quantitative measurement results; 2D-SWE, 2-dimensional shear wave elastography; Diam, diameter; Max, maximum value; Mean, mean value; Min, minimum value; SD, standard deviation.

We completed the 2D-SWE examination of the penile tissue in 20 rats. 2D-SWE images of each rat with suitable image quality were selected for SWQ measurement. The standard of a qualified 2D-SWE image (**Figure 1**) was determined as follows: the area of penile tissue was filled with color, similar to an oil painting without mosaic-like points. Results of the penile tissue SWQ value (9.10 ± 1.63 kPa) in each rat are shown in **Table 1**.

**Table 1.** SWQ, PAP_S_, and PAP_C_ measurements in rat penile tissue

**Number**	**Section**	**SWQ (kPa)**	**PAP_S_ (%)**	**PAP_C_ (%)**
1	NG	6.90	16.11	24.56
	M	6.90	14.76	24.45
	NT	7.00	14.57	25.01
2	NG	9.40	12.75	22.09
	M	5.80	17.15	27.64
	NT	9.50	13.17	19.42
3	NG	12.50	8.99	9.16
	M	8.90	8.13	8.11
	NT	8.50	13.49	7.11
4	NG	7.50	19.89	14.20
	M	5.90	28.84	20.28
	NT	5.70	28.79	18.18
5	NG	8.40	17.82	10.61
	M	8.40	12.93	12.11
	NT	8.30	7.73	18.71
6	NG	8.60	16.31	11.96
	M	7.60	18.83	15.77
	NT	7.70	20.17	18.33
7	NG	6.90	30.08	34.32
	M	7.00	38.75	29.57
	NT	5.60	33.72	31.44
8	NG	9.80	30.95	12.90
	M	9.80	21.58	10.42
	NT	12.60	14.02	12.80
9	NG	8.50	13.96	23.67
	M	8.20	10.52	26.86
	NT	9.60	11.61	27.99
10	NG	10.50	8.98	8.56
	M	11.10	9.93	11.21
	NT	10.40	11.62	8.89
11	NG	9.60	4.99	3.87
	M	8.80	4.06	4.03
	NT	7.30	4.16	4.72
12	NG	10.00	2.39	2.37
	M	9.90	2.76	2.09
	NT	10.40	2.72	1.64
13	NG	11.20	3.32	2.48
	M	10.60	3.33	2.47
	NT	9.70	3.70	3.92
14	NG	9.30	2.51	2.36
	M	10.20	1.93	2.81
	NT	10.30	1.64	2.64
15	NG	11.40	3.33	1.05
	M	9.60	3.88	1.21
	NT	8.80	4.31	1.60
16	NG	10.50	2.49	2.64
	M	9.10	3.61	3.15
	NT	10.20	3.47	3.55
17	NG	9.80	4.74	2.20
	M	10.70	2.14	2.65
	NT	8.50	4.17	3.15
18	NG	10.20	3.31	3.17
	M	9.40	4.19	3.59
	NT	9.00	4.20	3.49
19	NG	8.90	4.03	4.09
	M	9.20	3.73	4.90
	NT	7.70	4.93	5.62
20	NG	10.30	5.04	3.37
	M	9.50	4.44	3.20
	NT	12.50	3.35	2.68

M, the middle segment of the penis; NT, near the root segment of the penis; NG, near the segment of the penis glans; PAP_C_, positive area proportion of type III collagen fibers; PAP_S_, positive area proportion of alpha-smooth muscle actin; SWQ, shear wave elastic quantitative measurement.

### Immunohistochemistry results

**Figure 2.**
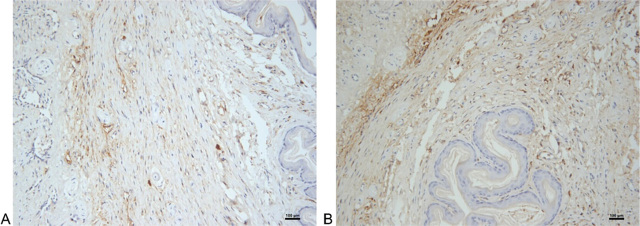
Expression of alpha-smooth muscle actin and type III collagen in rat penile tissue (× 200). Immunohistochemical staining showed positive results (brown areas). **(A)**: Alpha-smooth muscle actin. **(B)**: type III collagen. Bars were 100 μm.

We performed the immunohistochemical staining of penile tissue sections from 20 rats successfully. **Figure 2** shows the expression of alpha-smooth muscle actin and type III collagen in the penile tissue. The positive areas were stained brown. The measurement of PAP_S_ (10.55% ± 9.13%) and PAP_C_ (10.62% ± 9.48%) in each rat penile tissue sample are shown in **Table 1**.

## Multiple linear regression results

**Figure 3.**
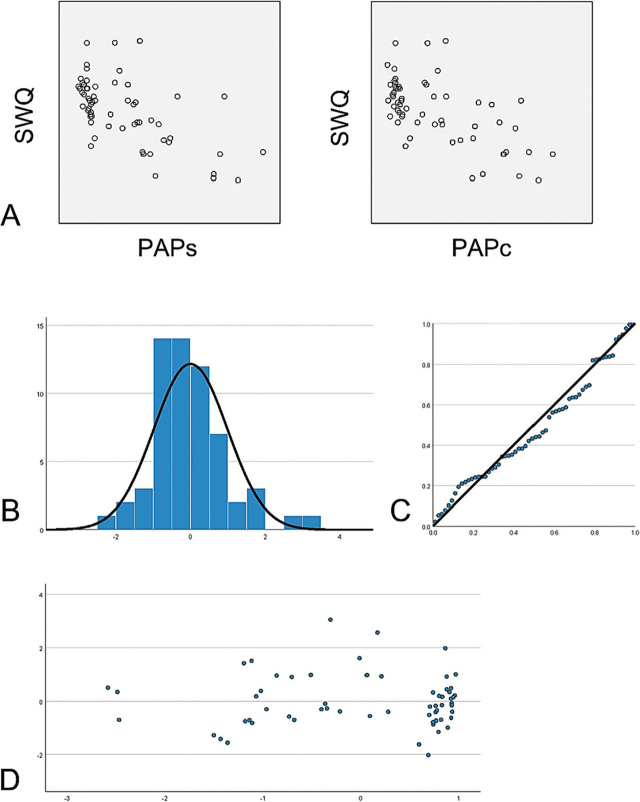
Multiple linear regression analysis results of **(A)** SWQ, PAP_S_, and PAP_C_. Scatter plots showed a linear correlation between SWQ and PAP_S_, SWQ, and PAP_C_, respectively. **(B)** The histogram and **(C)** P-P plot showed that the regression standardized residuals of SWQ were normally distributed (mean = 2.37E-15, SD = 0.983). **(D)** Residual plot showed that the regression standardized residuals distributed symmetrically around the 0-value, possessing independence and equal variance. SWQ, shear wave elastic quantitative measurement; PAP_S_, positive area proportion of alpha-smooth muscle actin; PAP_C_, positive area proportion of type III collagen fiber.

According to the data shown in **Table 1**, we found that SWQ followed a normal distribution (*P* = 0.282), but PAP_S_ and PAP_C_ did not (*P* < 0.001). Scatter plots (**Figure 3A**) showed that SWQ was linearly correlated with PAP_S_ and PAP_C_, respectively. There was no multicollinearity between PAP_S_ and PAP_C_ (tolerance = 0.385, VIF = 2.595). The Durbin–Watson statistic (= 1.418) and residual plots (**Figures 3B–D**) showed that residuals possessed normality, independence, and equal variance. Multiple linear regression results (**Table 2**) showed that the regression equation of the PAP_S_, PAP_C_, and SWQ (SWQ = 10.376 – 0.05 PAP_S_ – 0.07 PAP_C_) was of statistical significance (*F* = 21.153, *P* < 0.001). The content of SMCs and CFs was negatively correlated with SWQ, affecting 42.6% of the total variation in SWQ (*R*^2^ = 0.426).

**Table 2.** Multiple linear regression results in the rat penis

**Variables**	**Coefficient**	**Standard error**	***t***	***P***	***F***	***R^2^***
PAP_S_	−0.050	0.029	−1.745	0.086	21.152^***^	0.426
PAP_C_	−0.070	0.028	−2.521	0.015		
Constant	10.376	0.255	40.663	0.000		

PAP_c_, positive area proportion of type III collagen fibers; PAP_s_, positive area proportion of alpha-smooth muscle actin.

*** *P* < 0.001, indicating that the regression equation was of statistical significance.

## Discussion

**Figure 4.**
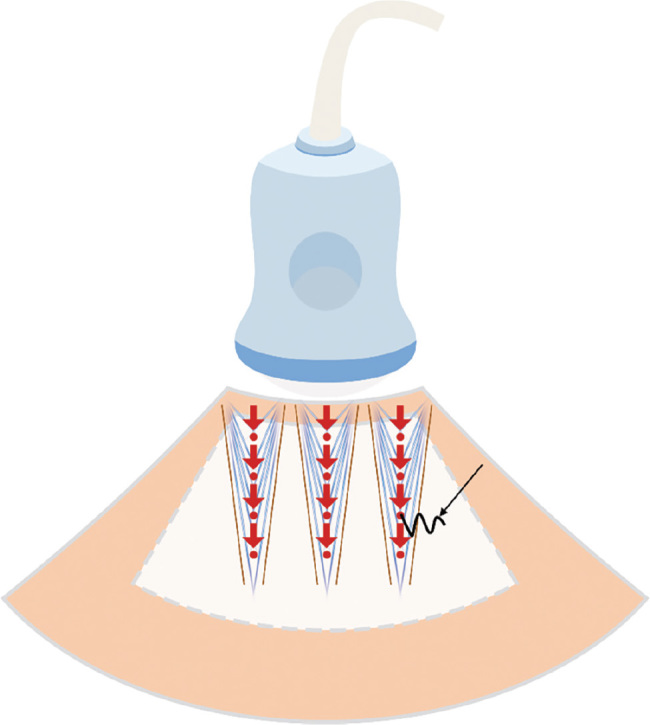
The principle of 2D-SWE. The probe emitted the exciting acoustic impulse (red thick arrows) to the measured tissue. The impulse continuously focused on the tissue, leading the particles (red circles) to vibrate. The shear wave (black arrow) formed and propagated transversely after tissue vibration. The shear wave velocity was measured to calculate the SWQ value reflecting the tissue elastic characteristics. The white area with dotted lines was the 2D-SWE imaging area (elastography map). 2D-SWE, 2-dimensional shear wave elastography; SWQ, shear wave elastic quantitative measurement.

2D-SWE is a new noninvasive ultrasound technology that has been successfully applied in clinical practice. The brief workflow of 2D-SWE (**Figure 4**) is as follows [1]: the exciting acoustic impulse emitted by the probe is continuously focused on the tissue to generate acoustic radiation force, which in turn leads to tissue vibration and shear wave formation. The shear wave propagating transversely in the tissue will be captured rapidly by the detected pulse. The SWQ value reflecting the tissue elastic characteristics can be calculated accordingly by measuring the shear wave velocity.

2D-SWE could quantitatively evaluate the tissue elastic characteristics and detect pathological changes in the tissue components by measuring the SWQ value. For example, 2D-SWE is available for follow-up and close monitoring in patients with kidney allograft fibrosis by assessing the degree of fibrosis, providing superiority over serum creatinine in the detection of early tubulointerstitial fibrosis [8]. 2D-SWE is also applied to distinguish benign from malignant breast lesions as the elastic characteristics of breast lesions correlate significantly with the content of CFs [9].

Besides, 2D-SWE is a reliable technique with high specificity and sensitivity for quantitative assessment of the elastic characteristics in the penile tissue [10, 11]. The SWQ value is correlated with the International Index of Erectile Function (IIEF-5) and Erection Hardness Scale (EHS) scores [11]. By measuring the penile SWQ value, 2D-SWE is able to detect and localize nonpalpable lesions in PD patients' curved penis undetected through conventional ultrasound [12], evaluate the degree of fibrosis in corpus cavernosum [13], differentiate the malignant from benign penile mass [14], and quantitatively evaluate the rigidity changes of corpus cavernosum in vascular ED patients [15]. Accurate assessment of the penile tissue composition seems imperative for the precise diagnosis of penile diseases. For example, pathological changes in penile tissue components will eventually result in organic ED. Timely and accurate assessment of the penile tissue components is the key to improving the accuracy of the diagnosis of ED.

Currently, penis biopsy and surgery are the only clinical methods for the assessment of penile tissue components. However, these methods are invasive and require high technical skills for the operators. Complications like penile pain, hematoma, fibrosis, scarring, and induration are prone to occur after invasive penile examinations, which seriously affect patients' physical and mental health. Thus, penis biopsy and surgery are not available as routine clinical examinations for patients with penile diseases. As the penis is a private external reproductive organ of the human body, patients always have difficulty accepting invasive examinations on it compared with other organs. For example, psychological pressure and potential complications will occur and aggravate the condition in ED patients after an invasive penile examination. Consequently, noninvasive penile examinations play an essential role in the diagnosis, treatment, and follow-up of patients with penile diseases. There is a critical need to explore a novel method for the noninvasive assessment of penile tissue components.

Studies have demonstrated that pathological changes in the component and content of penile tissue would affect the SWQ value measured by 2D-SWE [16]. Identifying the main factors affecting the penile SWQ value is the prerequisite for accurate diagnosis of the penile disease using 2D-SWE. Given that no relevant studies have been reported, this study was performed to analyze the main factors affecting the penile SWQ value.

SMCs (40%–52% in humans) and CFs (40%–65% in humans) are the major components of the penile tissue and the main components involved in erectile function [4, 5]. SMCs relaxation is the primary process of penile erection. CFs are essential components involved in penile stretching. During the process of penile erection, CFs prevent overstretching of the basement membrane and SMCs and protect the endothelial and underlying cell layers from disruption [17]. Furthermore, as components of the intracavernosal fibrous framework, CFs ensure normal erection, prevent the penis from deforming and bending during erection, and form a fibrous sheath to prevent arterial collapse and nerve compression [18]. Type III CFs are involved in the process of contraction during wound healing. Increased CFs content would result in plaque contracture and penile deformity. In theory, the composition and content of the tissue through which shear wave passes could directly affect the SWQ value. Therefore, the hypothesis of this study was that the content of SMCs and CFs may be the main factors affecting changes in penile SWQ.

In this study, our results showed that the regression equation of the SWQ with the PAP_S_ and PAP_C_ was statistically significant. The content of SMCs and CFs was one of the main factors affecting the penile SWQ value, which verified the hypothesis we proposed.

Although our data demonstrated that the content of SMCs and CFs was negatively correlated with SWQ, there were several limitations in this study. First, PAP_S_ and PAP_C_ did not follow a normal distribution. The small sample size may attribute to it. Second, the regression degree of the model (*R*^2^ < 0.5) was low, which indicated that there were other factors affecting the penile SWQ that need to be explored. An exploratory study in a new field might contribute to the reason why the *P* value of PAPs (*P* = 0.086) in the regression equation was more than 0.05 but smaller than 0.1. Additional research on a large amount of clinical data is needed before 2D-SWE can be put into application confidently.

## Conclusion

SMCs and CFs are the main factors affecting the SWQ value and are the primary tissue components determining the SWQ when quantitatively evaluating the elastic characteristics of penile tissue with 2D-SWE.

## References

[1] Shiina T, Nightingale KR, Palmeri ML, Hall TJ, Bamber JC, Barr RG (2015). WFUMB guidelines and recommendations for clinical use of ultrasound elastography: Part 1: basic principles and terminology. Ultrasound Med Biol..

[2] Cui A, Xu L, Mu J, Tong M, Peng C, Wu T (2018). The role of shear wave elastography on evaluation of the rigidity changes of corpus cavernosum penis in venogenic erectile dysfunction. Eur J Radiol..

[3] Lee JY, Jung DC, Lee S, Kang NG, Oh YT, Han K (2021). Stiffness of the central corpus cavernosum on shear-wave elastography is inversely correlated with the penile rigidity score in patients with erectile dysfunction. World J Mens Health..

[4] Wespes E, Goes PM, Schiffmann S, Depierreux M, Vanderhaeghen JJ, Schulman CC (1991). Computerized analysis of smooth muscle fibers in potent and impotent patients. J Urol..

[5] Lin JS, Tsai YS, Lin YM, Lin CS, Chow NH (2001). Age-associated changes in collagen content and its subtypes within rat corpora cavernosa with computerized histomorphometric analysis. Urology..

[6] Abidu-Figueiredo M, Ribeiro IC, Chagas MA, Cardoso LE, Costa WS, Sampaio FJ (2011). The penis in diabetes: structural analysis of connective tissue and smooth muscle alterations in a rabbit model. BJU Int..

[7] Percie du Sert N, Hurst V, Ahluwalia A, Alam S, Avey MT, Baker M (2020). The ARRIVE guidelines 2.0: updated guidelines for reporting animal research. PLoS Biol..

[8] Ma MK, Law HK, Tse KS, Chan KW, Chan GC, Yap DY (2018). Non-invasive assessment of kidney allograft fibrosis with shear wave elastography: a radiological-pathological correlation analysis. Int J Urol..

[9] Wang ZL, Sun L, Li Y, Li N (2015). Relationship between elasticity and collagen fiber content in breast disease: a preliminary report. Ultrasonics..

[10] Aybar MD, Turna O (2022). Assessment of the rigidity changes of corpus cavernosum penis in vasculary erectile dysfunction (ED) subtypes by shear wave elastography (SWE). J Ultrasound Med..

[11] Illiano E, Trama F, Ruffo A, Romeo G, Riccardo F, Iacono F, Costantini E (2021). Shear wave elastography as a new, non-invasive diagnostic modality for the diagnosis of penile elasticity: a prospective multicenter study. Ther Adv Urol..

[12] Richards G, Goldenberg E, Pek H, Gilbert BR (2014). Penile sonoelastography for the localization of a non-palpable, non-sonographically visualized lesion in a patient with penile curvature from Peyronie's disease. J Sex Med..

[13] Hamidi N, Altinbas NK, Gokce MI, Suer E, Yagci C, Baltaci S, Turkolmez K (2017). Preliminary results of a new tool to evaluate cavernous body fibrosis following radical prostatectomy: penile elastography. Andrology..

[14] Alis DC, Ustabasioglu FE, Samancý C, Boz SM, Sirolu S, Kantarcý F (2016). Penile masses: shear wave elastography correlated with magnetic resonance imagining. A two cases report. Med Ultrason..

[15] Zhou W, Zhang Y, Li L, Gao J, Zheng H, Huang M (2021). Evaluation of arterial erectile dysfunction using shear wave elastography: a feasibility study. J Ultrasound Med..

[16] Cheng H, Liu GX, Wang F, Wang K, Ruan LT, Yang L (2022). Quantitative assessment of the aging corpus cavernosum by shear wave elastography. Asian J Androl..

[17] Pinheiro AC, Costa WS, Cardoso LE, Sampaio FJ (2000). Organization and relative content of smooth muscle cells, collagen and elastic fibers in the corpus cavernosum of rat penis. J Urol..

[18] Goldstein AM, Meehan JP, Morrow JW, Buckley PA, Rogers FA (1985). The fibrous skeleton of the corpora cavernosa and its probable function in the mechanism of erection. Br J Urol..

